# Machine learning for predicting successful extubation in patients receiving mechanical ventilation

**DOI:** 10.3389/fmed.2022.961252

**Published:** 2022-08-11

**Authors:** Yutaka Igarashi, Kei Ogawa, Kan Nishimura, Shuichiro Osawa, Hayato Ohwada, Shoji Yokobori

**Affiliations:** ^1^Department of Emergency and Critical Care Medicine, Nippon Medical School, Tokyo, Japan; ^2^Department of Industrial Administration, Tokyo University of Science, Chiba, Japan

**Keywords:** extubation, intensive care unit, machine learning, mechanical ventilation, ventilator

## Abstract

Ventilator liberation is one of the most critical decisions in the intensive care unit; however, prediction of extubation failure is difficult, and the proportion thereof remains high. Machine learning can potentially provide a breakthrough in the prediction of extubation success. A total of seven studies on the prediction of extubation success using machine learning have been published. These machine learning models were developed using data from electronic health records, 8–78 features, and algorithms such as artificial neural network, LightGBM, and XGBoost. Sensitivity ranged from 0.64 to 0.96, specificity ranged from 0.73 to 0.85, and area under the receiver operating characteristic curve ranged from 0.70 to 0.98. The features deemed most important included duration of mechanical ventilation, PaO_2_, blood urea nitrogen, heart rate, and Glasgow Coma Scale score. Although the studies had limitations, prediction of extubation success by machine learning has the potential to be a powerful tool. Further studies are needed to assess whether machine learning prediction reduces the incidence of extubation failure or prolongs the duration of ventilator use, thereby increasing tracheostomy and ventilator-related complications and mortality.

## Introduction

Invasive mechanical ventilation is an essential component of invasive care. The number of patients receiving ventilator care has been reported to be 270–314 per 100,000 population per year (1), and this number has increased dramatically due to coronavirus disease 2019 (COVID-19) pneumonia. Approximately half of the patients on ventilators died (2), and more than one million people have died of COVID-19 in the US (3).

Ventilator liberation comprises one of the most critical decisions in the intensive care unit (ICU). If ventilated patients are extubated too early, they may require reintubation, which prolongs hospital stay and increases mortality (4–6). On the other hand, longer ventilation increases complications such as ventilator-associated pneumonia and mortality; thus, it is critical to determine the optimal timing of extubation for patients receiving ventilation (7). Various predictors and protocols have been used to predict successful extubation (8), but single factors are not accurate (9, 10). Although the use of protocols has reduced the duration of ventilator management and ICU stay (11), the proportion of extubation failure remains ≥10% (12). There is no standardized protocol for extubation, and each facility has its own criteria (13).

In recent years, electronic health records have been collected in ICUs, and these data are publicly available. Simultaneously, the application of machine learning in the medical field has rapidly progressed, making it possible to make various predictions in real time regarding patients in ICUs (14). Machine learning can potentially be a breakthrough in the prediction of extubation success, for which many factors are involved in a complex manner. To date, several papers have been published on the prediction of extubation outcomes using machine learning. In this mini-review, the methods and results of previous studies are summarized, and current issues and future directions have been reviewed.

## Protocols for scheduled extubation

Patients receiving ventilation are evaluated for extubation when the primary disease has improved, oxygenation and hemodynamics are stable, and the patient is breathing spontaneously and normally. A spontaneous breathing trial (SBT) is performed to assess whether the patient can tolerate minimal ventilatory support (15). SBT is performed with oxygenation <50%, low positive end-expiratory pressure (PEEP) (e.g., ≤ 5 cmH_2_O), and low PS (e.g., ≤ 5 cmH_2_O) for a certain period of time, and if there are no abnormalities in circulation or respiration, the patient is deemed to be ready for extubation. If the patient has upper airway problems (post upper airway surgery, positive cuff-leak test, history of difficult intubation), steroids may be administered before extubation (16). If the patient is at high risk for respiratory failure (chronic obstructive pulmonary disease, chronic respiratory failure, obesity, overhydration), prophylactic non-invasive positive pressure ventilation (non-invasive positive pressure ventilation and nasal high-flow) should be prepared after extubation.

Thus, during SBT and weaning, vital signs related to respiration and circulation (blood pressure, pulse rate, respiratory rate, SpO_2_, and boosting agent levels), arterial blood gas tests (PaO_2_, pH), and ventilator measurements (tidal volume, minute volume, and rapid shallow breathing index) have been used as criteria for extubation. Since all machine learning studies are backward-looking studies, the data are, in principle, obtained by performing SBT. Therefore, the process of performing SBT and that of determining extubation from the collected data remains the same when predicting extubation by machine learning.

## Aim of machine learning prediction

The most important goal of machine learning is to improve the prediction of the success or failure of extubation. In particular, it is expected that machine learning models can predict extubation failure and thereby reduce its incidence. Conversely, if a machine learning model is too conservative, in order to reduce the number of extubation failures, the period of ventilator management may be prolonged and tracheotomies may increase. Therefore, it is important to maintain a balance between these two factors.

Further, in some machine learning models, feature importance may represent objective indicators to explain the model. It is possible to compare the important features with the relevant guidelines to ensure that there is validity in the model's explanation. It is also possible to identify unknown factors that have not been used to determine extubation but are of high importance.

However, machine learning models cannot predict the causes of extubation failure, although reasons for extubation failure, such as laryngeal edema, are important. The purpose of machine learning models is not to predict the causes of extubation failure but to support decision-making.

## Methods of previous studies

A total of seven studies on the prediction of extubation using machine learning were published between 2015 and 2021 (Table 1). We compared the differences between the methods of each study; studies on ventilator weaning were excluded (24).

**Table 1:** Summary of the seven studies.

**Author, year**	**Algorithm with the highest accuracy**	**Dataset (Training and test) (N, patients)**	**Dataset (External validation) (N, patients)**	**Number of features**	**Definition and rate of extubation failure**	**n-fold cross-validation**	**Accuracy**	**Sensitivity**	**Precision**	**Specificity**	**F1 score**	**AUROC**	**Strengths**	**Weakness**
Kuo (17)	ANN	Two hospitals (*N* = 121)	No	8	<48 h, 26%	5	0.80	0.82	N/A	0.73	N/A	N/A	To determine the optimal number of hidden-layer perceptron, it was set from 10 to 39. Balanced data because there was failed extubation in 26% of patients.	Small number of cases. Fewest predictors: vital signs and laboratory results were not used. Undersampling and external validation were not performed.
Hsieh (18)	ANN	Single hospital (*N* = 3,602)	No	37	<72 h, 5%	10	N/A	0.822	0.939	N/A	0.867	0.85	Better prediction compared with other weaning parameters.	Undersampling was not performed, although there was failed extubation in only 5% of the patients. External validation was not performed.
Chen et al. (19)	LightGBM	MIMIC-III (*N* = 3,636)	No	68	<48 h, 17%	5	0.8020	0.8394	N/A	0.7477	N/A	0.8198	After developing a model with all features, the model was created again with only the important features. The synthetic minority oversampling technique (SMOTE) was used, but results with or without SMOTE were not obvious. Feature importance and SHAP value were obtained.	External validation was not performed.
Fabregat (20)	SVM	Single hospital (*N* = 1,108)	No	19	<48 h, 9%	7	0.946	N/A	N/A	N/A	N/A	0.983	Highest accuracy and AUROC. Undersampling was performed.	Although having the highest accuracy and AUROC, data from the same patient was included in both training and test data sets, making cheating possible. External validation is required. Laboratory results were not used for prediction. Predictive performance was not obtained without accuracy and AUROC.
Otaguro (21)	LightGBM	Single hospital (*N* = 117)	No	58	<72 h, 11%	5	0.9265	0.9602	0.9146	N/A	0.9369	0.9502	Undersampling was performed. Feature importance was obtained.	Small number of cases. Lowest precision, but more than 0.90. Although having the highest sensitivity, data from the same patient were included in both training and test data sets, making cheating possible.
Zhao et al. (22)	CatBoost	MIMIC-IV (*N* = 16,189)	Single hospital (*N* = 502)	78	<48 h, 17^a^ and 11%^b^	N/A	N/A	0.64	0.97	0.85	0.77	0.80	Highest specificity and precision. Largest number of cases and features. Clinical scores were not used because they make the models inconvenient in clinical settings. Eleven models were developed and compared with other predictive factors commonly used in the ICU. External validation was performed. Feature importance and SHAP value were obtained.	Lowest sensitivity and F1 score. Clinical scores were commonly used for developing models, but this study did not use them.
Fleuren (23)	XGBoost	COVID-19 database (*N* = 883)	No	20	<48 h, 13%; <7 days 19%	5	N/A	N/A	N/A	N/A	N/A	0.70	The most detailed information on sedative and analgesic dosages. SHAP value was obtained.	Lowest AUROC. Predictive performance was not obtained without AUROC. Not generalizable because it only involved patients with COVID-19. External validation was not performed.

*ANN, artificial neural network; AUROC, area under the receiver operating characteristic curve; COVID, coronavirus disease; MIMIC, Medical Information Mart for Intensive Care; N/A, not available; SHAP, shapley additive explanations; SVM, support vector machine. Because Chen and Zhao's study was about extubation failure prediction, the sensitivity and specificity of the original data were replaced.*

a *Training and test dataset,*

b *External validation dataset*.

### Dataset

Some studies used single-center ICU databases with hundreds to thousands of cases, while others used nationwide databases for COVID-19 or large free-access databases, with thousands to tens of thousands of cases. For example, the Medical Information Mart for Intensive Care (MIMIC) is a large, single-center, free-access database comprising information relating to patients admitted to the ICU of a large tertiary care hospital with a variety of diseases, such as myocardial infarction, postoperative complications, medical complications, and trauma (25). Machine learning using such a database may be adaptable to patients with a wide variety of diseases.

The dataset is divided into training data for model development and test data for evaluation. If multiple data from one patient are used to increase the sample size, data from the same patient may be included in both training and test data. If a test is performed on the data of the patient for whom the model was developed, it will be a so-called cheat, resulting in a higher than actual accuracy. Therefore, one patient's data must be used in either the training or test data.

### Features

The features used for prediction can be categorized as follows: demographic information, vital signs, laboratory results, ventilator information, clinical intervention, and clinical scores (Table 2). Features that are clinically important and are frequently used in clinical practice should be included in the protocols.

**Table 2:** Classification and listing of features.

**Features**	**Kuo**	**Hsieh**	**Chen**	**Fabregat**	**Otaguro**	**Zhao**	**Fleuren**
**Demographic**							
Age	X	X	X	X	X	X	X
Gender		X	(X)	X	X	X	X
Ethnicity						X	
Weight			X		X		
Height			X		X		
Body mass index		X		X	X	X	X
Weight loss			(X)				
Past medical history		X	(X)			X	X
Charlson index						X	
Reasons for respiratory failure	X						
**Vital signs**							
Heart rate		X	X	X	X	X	X
Respiratory rate		X	X	X	X	X	X
Body temperature			X		X	X	
Systolic blood pressure			X		X		
Diastolic blood pressure			X		X		
Mean arterial pressure		X	X		X	X	
Glasgow coma scale			X	X	X	X	X
Richmond agitation-sedation scale			X	X			X
SpO_2_				X		X	
O_2_ saturation to inspired fraction ratio				X		X	
SpFiO2/RR				X			
End-tidal carbon dioxide					X		
Number of premature ventricular contraction					X		
**Laboratory results**							
White blood cell			X		X	X	X
Red blood cell					X	X	
Hemoglobin		X	X		X	X	
Hematocrit		X			X	X	X
Platelet			X		X	X	X
Mean corpuscular volume						X	
Mean corpuscular hemoglobin						X	
Mmean corpuscular hemoglobin concentration						X	
Red cell distribution width						X	
Arterial pH		X	X		X	X	
PaCO_2_		X	X		X	X	X
PaO_2_		X	X		X	X	
P/F ratio		X			X	X	X
SaO2			X		X	X	
Base excess					X	X	
Na^+^		X	X		X	X	
K^+^		X	X		X	X	
Ca^+^		X	X		X	X	
P^+^		X					
Cl^−^		X	X		X	X	
HCO3^−^					X	X	
Anion gap					X	X	
Lactate			X		X	X	
Carboxyhemoglobin					X		
Methemoglobin					X	X	
Alveolar-arterial oxygen gradient			(X)				
Central venous oxygen saturation			X			X	X
Glucose		X	X			X	X
Creatinine		X	X		X	X	
Blood urea nitrogen (BUN)			(X)				
Troponin			(X)		X		
Total protein			(X)				
B-type natriuretic peptide			(X)		X		X
C-reactive protein			(X)		X		
Aspartate aminotransferase			(X)		X	X	
Alanine transaminase					X	X	
Lactate dehydrogenase (LDH)					X	X	
Alkaline phosphatase					X		
Creatine phosphokinase			(X)		X	X	
Total bilirubin		X	X		X	X	
Albumin					X		
Amylase			(X)			X	
Prothrombin time			X		X	X	
Activated partial thromboplastin time			X		X	X	
PT/INR					X	X	
Fibrinogen							
**Ventilator information**							
Number of previous mechanical ventilation events				X			
Time under mechanical ventilation (T_MV_)	X	X	X	X	X	X	X
Hours since last controlled mode							X
Ventilation mode				X			
Fraction of inspired oxygen		X			X	X	X
Tidal volume	X		X	X	X	X	
Tidal volume per kg ideal body weight							X
Minute volume		X	X		X		
Mean airway pressure			X		X	X	
Peak inspiration pressure			X	X	X		
Plateau pressure				X		X	
PEEP		X				X	X
Positive end-expiratory pressure		X			X	X	X
Maximum inspiratory pressure		X					
Maximum expiratory pressure		X					
Airway occlusion pressure							X
Ventilatory ratio							X
Inspiratory time	X						
Expiratory time	X						
Spontaneous breathing trial success times						X	
**Clinical intervention**							
Hospital stay	X						
ICU stay	X						
Sedation day			X				
Sedatives and analgesics dose				X			X
Total cumulative dose (sedatives and analgesics)							X
Vasopressor			(X)			X	
Antibiotic type (ABX)						X	
Fluid balance							X
Urine output						X	
Continuous renal replacement therapy						X	
Crystalloid and colloid amount						X	
Transufusion (RBC, FFP, PLT)						X	
Hours since last proning session						X	
Central venous pressure						X	
Rapid shallow breathing index		X	(X)	X		X	
**Clinical scores**							
Sequential organ failure assessment			X			X	
Simplified acute physiology score = =						X	
Acute physiology and chronic health evaluation-II	X			X			X
SEMICYUC code				X			
ROX index						X	
**Top 5 important features**							
1	N/A	N/A	T_MV_	N/A	T_MV_	Strokes	N/A
2			PaO2		Age	RR	
3			PaCO2		PEEP	ABX	
4			pH		LDH	T_MV_	
5			BUN		APTT	SpO2	

*FiO2, fraction of inspired oxygen; PT/INR, Prothrombin time and international normalized ratio; N/A, not applicable; P/F, arterial oxygen partial pressure to fractional inspired oxygen; ROX, respiratory rate-oxygenation; SEMICYUC, Sociedad Española de Medicina Intensiva, Crítica y Unidades Coronarias; SpFiO2/RR, respiratory rate-oxygen index. Sedatives and analgesics include benzodiazepine, clonidine, dexmedetomidine, fentanyl, haloperidol, midazolam, propofol, and quetiapine. (X) was included in the first 68 features but was not in the top 36, so it was not used to develop the second model*.

The largest number of features that have been used for the prediction of extubation success thus far is 78. Using a larger number of features may result in more accurate predictions and may help identify unknown features that affect predictions. On the other hand, as the number of features increases, the proportion of missing values also increases because they may not have been measured; if this proportion exceeds a certain percentage, the missing values are excluded from the list of features. Missing values can be compensated for to a certain extent, but an increase in the number of missing values affects the development and prediction of the machine learning model. Some studies have developed models using all possible features, as well as new models using only features of high importance. For example, in the study by Chen et al. (19) features with missing data of ≥40% were not used, and missing values were compensated for by using the average values of the other patients. The model was initially developed with 68 features, and finally, a prediction model was developed with 36 of the most important features. A comparison of the accuracy based on the number of features using LightGBM, which had the highest accuracy, revealed that the accuracy ranged from 0.8023 to 0.8020, sensitivity from 0.7485 to 0.7477, specificity from 0.8327 to 0.8394, and area under the curve from 0.8130 to 0.8198; all these values were almost equivalent.

### Outcomes (labeling)

Supervised learning involves labeling the correct answer (success or failure), called annotation. Failed extubation was defined as reintubation within 48 or 72 h of extubation in previous studies. The proportion of extubation failure ranged from 5 to 26%.

In general, most studies on ventilator liberation define extubation failure as reintubation within 24–72 h (26, 27), or up to 7 days (28, 29). It is difficult to define patients who require non-invasive positive pressure ventilation (NPPV) or nasal high-flow as successful extubations. However, studies have shown that post-extubation NPPV or nasal high-flow reduces reintubation compared to standard oxygen therapy, and clinical practice guidelines also recommend nasal high-flow for high-risk patients (30–34). Therefore, the use of NPPV or nasal high flow cannot be defined as extubation failure as they are sometimes used routinely.

### Machine learning algorithms

In general, artificial neural networks are models that mimic parts of the neural circuits of the brain, which are highly accurate but cannot be explained, like a black box. On the other hand, since medicine emphasizes causal relationships and accountability to patients, models that can be explained by the importance of features, Shapley additive explanations (SHAP) values, and decision trees are sometimes preferred. This method is often used to improve performance by creating multiple weak models (weak learners), called boosting, where the previous learner is repeatedly modified by the next learner.

Predictive models were built using multiple algorithms and compared for accuracy. No model was consistently accurate, and results varied across the studies. It is difficult to determine which algorithm is most appropriate by comparing the values of accuracy across studies because some studies do not list all scores.

### Validation

When machine learning models are developed at a single institution or with a small number of cases, there is a possibility that biased models will be created. Therefore, it is necessary to perform external validation to evaluate the accuracy using data that were not used for model construction. By evaluating the accuracy using external data, it is possible to know if the machine learning model is generalizable. However, only one study conducted external validation (22).

### Performance evaluation

The common performance metrics used to evaluate the predictive performance of the model are accuracy, sensitivity (recall), precision (positive predictive value), specificity, and F1 score. In the equations given below, successful extubation is described as positive and failed extubation as negative. Note that in the opposite case, sensitivity and specificity are opposite (Table 1).

Accuracy is the ratio of correct predictions to the total number of predictions.


$$\begin{array}{l} \text{Accuracy}=\left(\text{TP}+\text{TN}\right)/\left(\text{TP}+\text{FP}+\text{FN}+\text{TN}\right) \end{array}$$

TP, true positive; TN, true negative; FP, false positive; FN, false negative.

Sensitivity (recall) is the ratio of true positives to total actual positives. It represents the proportion of patients who underwent successful extubation and in whom extubation was predicted to be successful.


$$\begin{array}{l} \text{Sensitivity}=\text{ TP}/\left(\text{TP}+\text{FN}\right) \end{array}$$

Precision (positive prediction value) is the ratio of true positives to total predicted positives.


$$\begin{array}{l} \text{Precision}=\text{ TP}/\left(\text{TP}+\text{FP}\right) \end{array}$$

Specificity is the ratio of true negatives to total negatives. It indicates the proportion of patients who underwent unsuccessful extubation and in whom extubation was predicted to be unsuccessful.


$$\begin{array}{l} \text{Specificity}=\text{TN}/\left(\text{FP}+\text{TN}\right)\\ \text{F}1-\text{score is the harmonic mean of precision and recall}.\\ \text{F}1\text{ score}=2\times \left(\text{recall}\times \text{precision}\right)/\left(\text{recall}+\text{precision}\right). \end{array}$$

Among the seven studies, sensitivity ranged from 0.64 to 0.96, precision ranged from 0.91 to 0.97, specificity ranged from 0.73 to 0.85, and the area under the receiver operating characteristic curve ranged from 0.70 to 0.98 (Table 1).

### Feature importance

Three studies showed the importance of features and two studies showed SHAP values. In the study by Chen et al., the top 10 most important features were duration of mechanical ventilation, PaO_2_, PaCO_2_, arterial pH, blood urea nitrogen (BUN), mean heart rate, weight, age, creatine, and Glasgow Coma Scale (GCS) score (19). In the study by Otaguro et al. (21) the top 10 most important features were duration of mechanical ventilation, age, PEEP, lactate dehydrogenase, activated partial thromboplastin time, alveolar arterial oxygen tension difference gradient, BUN, GCS score, C-reactive protein (CRP), and albumin. In the study by Zhao et al. (22) the top 10 most important features were stroke, respiratory rate, antibiotic type, pressure support ventilation level, central venous pressure, mechanical ventilation duration, SpO_2_, PEEP, heart rate, and number of successful SBTs.

Mechanical ventilation duration was included in three studies; PaO_2_ (or SpO_2_), BUN, heart rate, GCS, age, and PEEP in two studies; BUN, creatinine, CRP, and albumin were not included in the protocols, but may have influenced the success or failure of extubation because these features reflect the patient's general condition (BUN and creatinine reflect renal function, CRP reflects inflammatory status, and albumin reflects nutritional status).

## Discussion

We reviewed studies that used machine learning to predict the success or failure of extubation. Protocols determine extubation based on whether a patient meets certain defined conditions, but machine learning makes predictions based on certain features. Currently, it remains unclear whether protocols or machine learning contributes to a higher extubation success rate.

### Performance evaluation

A model with high specificity is preferable for predicting extubation failure. On the other hand, there exists a trade-off relationship: the higher the specificity, the lower the sensitivity. Therefore, the number of patients in which extubation is actually possible but expected to fail increases. This increases the risk of prolonging the duration of ventilator management and increasing tracheostomies.

For example, Zhao's study has the highest specificity of 0.85 but the lowest sensitivity of 0.64. Therefore, this model was able to predict the highest number of patients who failed extubation, so if this model predicts successful extubation, the probability of successful extubation is quite high, with a precision of 0.97. This model could reduce the rate of extubation failure from its current value of 11% to a theoretical 3%. In contrast, a model with high sensitivity can predict more patients who will succeed extubation. However, this model cannot be used for monitoring for possible early extubation. These models were trained on patients who underwent SBT and were deemed extubatable, and did not include patients whose final decision was not to be extubated. As a result, these models cannot be automatically used for all patients in the ICU to alert the timing of extubation, and must be considered as a support tool aimed to validate the physician's decision upon extubation. As the sensitivity increases, the specificity decreases, which leads to an aggressive model that predicts success in patients who cannot be extubated. A mode with high sensitivity and low specigicity is not suitable for this purpose. Therefore, a model with high specificity that can predict as many extubation failures as possible is preferred. If a conservative model for extubation is used in clinical practice, it is important to use the neative predictive valie as an indicator to avoid prolonged ventilatory management.

### Problems with machine learning methods

The Transparent Reporting of a multivariable prediction model for Individual Prognosis Or Diagnosis (TRIPOD) serves as a guideline for the development, validation, and update of clinical prediction models (35). The TRIPOD statement aims to increase the transparency of reporting on prediction models, regardless of the research methodology. We believe that all studies on prediction models using machine learning should be reported according to the TRIPOD checklist.

Several of the reviewed studies included issues related to imbalanced data and validation. In many databases, the failure rate of extubation was 10–20%. The number of successful and failed extubations was 9:1 to 8:2 in imbalanced data, and even if there was a model that predicted all successful extubations, it would have an accuracy of 80–90%, which could lead to a model that excessively predicts successful extubations. In this case, balancing the number of samples by under or oversampling can prevent the model from being overly predictive of successful extubation. Under or oversampling should be done in models that predict successful or failed extubation because the dataset is likely to be unbalanced. Some of the studies used data from multiple time points prior to extubation to increase the sample size for extubation failure, whereas some used patient data that were used as training data during cross validation as test data, which may have resulted in a higher than actual accuracy. External validation should be performed on databases that were not used as training data. In a single-center dataset, the unique extubation process of the institution may have influenced the development of the machine learning model. ICU datasets are freely available [Amsterdam UMCdb (36), eICU-CRD (37), HiRID (38), and MIMIC-IV (25)]; it is possible to perform external validation using these datasets. Conversely, it is also possible to build a machine learning model using these datasets and perform external validation using the dataset of a single institution.

### Future prospects

All the studies reviewed here were retrospective, and no prospective studies have been conducted thus far. It is necessary to evaluate the accuracy of machine learning via prospective studies and to compare it with the physicians' decisions. However, since it is ethically questionable to make an extubation decision based solely on the results of machine learning, it is necessary to evaluate the predictions and outcomes of machine learning when the physician makes the decision to extubate, assuming that the results of machine learning are blinded to the physician. Furthermore, it is necessary to consider how synergy between using the protocol and the predictions of machine learning can be obtained.

Finally, to verify whether machine learning predictions are useful in clinical practice, a study is required in which a group of patients are randomly divided into two groups: those who underwent extubation using machine learning predictions and those who underwent extubation without using machine learning predictions. Even if machine learning reduces the incidence of extubation failure, outcomes need to be evaluated to assess whether it prolongs the duration of ventilator use and increases performance of tracheostomies, ventilator-related complications, and mortality.

## Author contributions

Conception and design: YI. Acquisition of data: YI, KO, KN, and SO. Analysis and interpretation of data and drafting or revising the article: YI, KO, KN, SO, HO, and SY. All authors contributed to the article and approved the submitted version.

## Funding

This work was supported by KAKENHI 20K17876, Grant-in-Aid for Young Scientists.

## Conflict of interest

The authors declare that the research was conducted in the absence of any commercial or financial relationships that could be construed as a potential conflict of interest.

## Publisher's note

All claims expressed in this article are solely those of the authors and do not necessarily represent those of their affiliated organizations, or those of the publisher, the editors and the reviewers. Any product that may be evaluated in this article, or claim that may be made by its manufacturer, is not guaranteed or endorsed by the publisher.
